# Dissecting the molecular mechanisms of T cell infiltration in psoriatic lesions *via* cell-cell communication and regulatory network analysis

**DOI:** 10.1515/biol-2025-1231

**Published:** 2025-12-31

**Authors:** Kexin Li, Xinhong Ge, Yuanyuan Shang, Yaning Jiao, Lingdi Dong, Yingyao Yu

**Affiliations:** Department of Dermatology, General Hospital of Ningxia Medical University, Yinchuan, China

**Keywords:** psoriasis, single-cell RNA sequencing, IL-6-STAT3 signaling, cucurbitacin E, midkine (MDK)

## Abstract

This study aims to elucidate the intercellular communication mechanisms underlying T cell infiltration in psoriatic skin. Single-cell RNA sequencing revealed increased proportions of endothelial cells and T cells, alongside a reduction in melanocytes in psoriatic skin. Pseudotime analysis demonstrated dynamic transitions in T cell states, with significant gene expression changes at key branching points. These genes were enriched in pathways related to the ribosome, cytoplasmic translation, and ribosomal structural components. Cell-cell communication analysis showed enhanced interactions, particularly between T cells and fibroblasts in psoriasis. Fibroblasts exhibited upregulated MK signaling, characterized by elevated MDK expression and increased MDK receptor levels on T cells, suggesting MDK-mediated T cell recruitment. Regulatory network analysis identified IL-6 as a primary ligand regulating MDK *via* STAT3 and AHR, with increased expression of IL-6R, STAT3, and AHR in psoriatic fibroblasts. CuE treatment significantly alleviated psoriasis-like symptoms in a mouse model, as evidenced by reduced PASI scores, epidermal hyperplasia, and inflammatory cytokine levels. Mechanistically, CuE inhibited the STAT3/MDK signaling pathway, indicating its role in modulating fibroblast-driven T cell recruitment in psoriatic inflammation. This study demonstrates that CuE alleviates psoriasis-like symptoms by inhibiting the IL-6-STAT3-MDK signaling axis, positioning it as a potential novel therapeutic targeting fibroblast-mediated immune interactions in psoriasis.

## 1 Introduction

Psoriasis is a chronic, immune-mediated inflammatory skin disorder that affects over 60 million adults and children globally [1]. Recognized by the World Health Organization (WHO) as a “chronic, non-communicable, painful, disfiguring, and disabling disease with no cure,” psoriasis impacts both men and women equally, with a mean age of onset at 33 years [2], 3]. The condition significantly reduces patients’ quality of life and imposes a considerable psychosocial burden [4], 5].

The pathogenesis of psoriasis is multifactorial, driven by a complex interplay of immune, genetic, and environmental factors. Immunologically, tumor necrosis factor (TNF) and Th17 immune responses, along with cytokines such as interleukin (IL)-23 and IL-17, are critical in disease initiation and progression [6], [7], [8]. Genome-wide association studies have identified multiple susceptibility genes predominantly involved in immune regulation, highlighting the pivotal role of immune dysregulation in psoriasis [9], [10], [11]. Environmental triggers, including psychological stress, infections, and skin trauma, can also activate inflammatory pathways in genetically predisposed individuals. Additionally, aberrant keratinocyte proliferation and differentiation, driven by the dysregulation of intracellular signaling pathways like NF-κB and JAK-STAT, are hallmark features of the disease [12], 13].

T cells play a central role in psoriasis pathophysiology. Interactions between skin-resident T cells and stromal cells initiate and perpetuate chronic inflammation, contributing to the formation of psoriatic plaques [14], 15]. Dermal T cell expansion correlates with disease severity and remission throughout the course of the disease [16]. Various T cell subsets, such as Th17 and Th22 cells, secrete pro-inflammatory cytokines that drive keratinocyte hyperproliferation and inflammation [17], 18]. Moreover, CD8^+^ T cells infiltrating psoriatic lesions release cytokines, including IL-17A, which exacerbate keratinocyte dysfunction and inflammatory responses [19], 20].

Recent advances highlight the critical role of cell-cell communication in psoriasis pathogenesis, involving both direct and indirect interactions between immune and non-immune cells within the skin microenvironment [21]. Functional interactions between T lymphocytes and keratinocytes have been shown to require direct cell contact to induce pathogenic effects [22]. Regulatory network analysis offers an essential framework for understanding the complex signaling events in psoriasis. Integrating experimental and computational approaches allows for a comprehensive mapping of signaling pathways and the identification of novel regulatory interactions, providing new insights into disease mechanisms and potential therapeutic targets.

In this study, single-cell transcriptomic analysis, pseudotime trajectory analysis, and cell-cell communication profiling were integrated to investigate the dynamic immune-stromal interactions in psoriatic lesions. Additionally, *in vivo* experiments were conducted to examine the regulatory role of the STAT3/MDK signaling axis in psoriatic inflammation, with the aim of identifying potential therapeutic strategies for psoriasis.

## 2 Materials and methods

### 2.1 Data source

The dataset used in this study (GSE173706) was obtained from the publicly available Gene Expression Omnibus (GEO) database (https://www.ncbi.nlm.nih.gov/geo/). Expression profile data were downloaded using the GEOquery R package (v2.72.0). This dataset consists of 33 single-cell RNA sequencing (scRNA-seq) samples. For analysis, eight samples from normal skin tissue and 14 samples from psoriatic skin tissue were selected.

### 2.2 Single-cell data analysis

scRNA-seq data from normal and psoriatic skin tissue samples in the GSE173706 dataset were used for analysis. The raw data were imported into the R environment using the DropletUtils package (v1.24.0). Cells were filtered based on sequencing depth (total counts >2000), the number of detected features, and the proportion of mitochondrial UMI counts relative to total UMI counts, in order to exclude low-quality cells and potential doublets. Data processing, including normalization, identification of highly variable genes, batch effect removal, dimensionality reduction, and clustering, was performed using the scran (v1.34.0), scater (v1.34.1), batchelor (v1.22.0), and bluster (v1.16.0) [7] R packages. Cell type annotation was subsequently conducted based on known marker genes.

### 2.3 Pseudotime analysis

Pseudotime analysis assumes that cells within the same tissue type exist in multiple continuous states at any given time, with transitions between states reflected by gene expression changes. By analyzing the distribution of gene expression profiles across individual cells, a trajectory of cellular state transitions can be inferred. In this study, Monocle (v2.32.0) was used to perform pseudotime analysis on T cells (CD4^+^ and CD8^+^ T cells) to explore their differentiation states in relation to disease and control samples. CD4^+^ and CD8^+^ T cells were extracted from the scRNA-seq dataset, and a Monocle cell dataset object was constructed using the newCellDataSet function. Data normalization, identification of highly variable genes, dimensionality reduction, and cell ordering along the pseudotime trajectory were then conducted.

### 2.4 Cell-cell communication analysis

Cell-cell communication analysis was performed using the CellChat package (v2.1.2) to investigate intercellular signaling differences between normal and psoriatic skin tissues. The overall number and strength of cell-cell interactions between the two groups were compared, and interaction strength distributions were visualized using scatter plots. Specific signaling pathways in fibroblasts with significant alterations in psoriatic tissue compared to normal skin were identified and visualized using dot plots.

### 2.5 GO and KEGG enrichment analyses

Gene Ontology (GO) and Kyoto Encyclopedia of Genes and Genomes (KEGG) are functional annotation databases that classify genes into sets based on shared biological functions or pathways. GO and KEGG enrichment analyses assess the overrepresentation of specific gene sets among differentially expressed genes compared to a background gene set using a hypergeometric test. This approach allows for the identification of biological processes or functions that differ significantly between experimental and control groups. In this study, GO and KEGG enrichment analyses of branch-specific differentially expressed genes were performed using the clusterProfiler R package (v4.14.4) to investigate functional alterations in T cells at trajectory branching points.

### 2.6 Regulatory network analysis

NicheNet is a computational framework designed to predict how intercellular communication influences gene expression. By integrating ligand-receptor interactions, intracellular signaling pathways, and transcriptional regulatory networks, NicheNet constructs detailed intercellular communication maps to identify ligands that may regulate the expression of target genes in recipient cells. In contrast to tools like CellChat and CellPhoneDB, which focus primarily on cell surface interactions, NicheNet also predicts downstream genes that may be regulated by these interactions. In this study, NicheNet was used to identify ligands in the cellular microenvironment that could regulate the expression of the target gene MDK in fibroblasts by modulating specific transcription factors.

### 2.7 Drug-gene interaction analysis

The Drug-Gene Interaction Database (DGIdb; https://dgidb.org/) is an integrated platform that compiles drug-gene interaction data from various resources, including DrugBank, PharmGKB, ChEMBL, and DrugTargetCommons. These interactions may involve drugs influencing gene expression by interacting with genomic DNA or modulating gene product activity. In this study, DGIdb was utilized to retrieve all predicted drug-gene interactions for the transcription factors associated with MDK. The resulting interaction network was visualized using network diagrams.

### 2.8 Animals and experimental design

All animal experiments were conducted in strict accordance with the Laboratory Animal Management Regulations of Ningxia Province. Male C57BL/6 mice, aged 6–8 weeks, were purchased from Beijing Vital River Laboratory Animal Technology Co., Ltd. (Beijing, China). Mice were randomly assigned to three groups: Control (*n* = 5), IMQ model (*n* = 5), and IMQ + CuE treatment (*n* = 5). To establish an acute psoriasis-like model, imiquimod (IMQ) cream (5 % concentration) was topically applied to the shaved dorsal skin once daily for seven consecutive days. Mice in the CuE treatment group received a daily intraperitoneal injection of Cucurbitacin E (CuE) at a dose of 10 mg/kg following IMQ application. The severity of psoriasis-like lesions was assessed on day 7 using the Psoriasis Area and Severity Index (PASI).

**Ethical approval:** The research related to animal use has been complied with all the relevant national regulations and institutional policies for the care and use of animals, and has been approved by the Ethics Committee of the General Hospital of Ningxia Medical University.

### 2.9 Hematoxylin and eosin (HE) staining

On day 7 post-treatment, the mice were euthanized *via* intraperitoneal anesthesia. A 15 mm^2^ square piece of skin tissue centered on the psoriatic lesion was excised from each mouse (*n* = 5 per group). The collected skin samples were fixed in 4 % paraformaldehyde at room temperature for 48 h, followed by standard dehydration, paraffin embedding, and sectioning. H&E staining was performed to examine the structural characteristics of psoriatic lesions, including epidermal thickness, granulation tissue formation, and inflammatory cell infiltration. Epidermal thickness was quantified from at least five randomly selected high-power fields per section.

### 2.10 Real-time fluorescence quantitative polymerase chain reaction (RT-qPCR)

Total RNA was extracted from dorsal skin samples (*n* = 5 per group) using a reagent from Thermo Fisher (USA), followed by reverse transcription using the SYBR Premix Ex Taq kit (Bao Biological Engineering, China) according to the manufacturer’s instructions. The reverse transcription reaction was performed under the following conditions: 95 °C for 10 min, followed by 40 cycles of 95 °C for 5 s, 60 °C for 30 s, and 70 °C for 60 s. Relative gene expression levels were quantified using the 2^−△△Ct^ method with ABI software (Foster City, CA).

STAT3: forward 5′-GAGAATCGTGGAGCTGTTTAG-3′, reverse 5′-GACCAGCAACCTGACTTTAG-3′; MDK: forward 5′-AAGGAGTTTGGAGCCGACTG-3′, reverse 5′-CATTGTAGCGCGCCTTCTTC-3′; GAPDH (internal control): forward 5′-tGTTCGTCATGGGTGTGAAC-3′, reverse 5′-TGAGTCCTTCCACGATACCA-3′.

### 2.11 Western blot analysis

Protein expression was analyzed by Western blot. Protein quantification was performed using the BCA protein concentration assay kit. Equal amounts of protein were separated by 10 % sodium dodecyl sulfate-polyacrylamide gel electrophoresis (SDS-PAGE), transferred to a nitrocellulose membrane, and blocked for 1 h with 5 % skimmed milk in Tris-buffered saline containing 0.1 % Tween-20 at room temperature. The membranes were incubated overnight at 4 °C with the following primary antibodies: IL-6 (1:1,000, 26404-1-AP, Proteintech, Wuhan, China); TNF-α (1:1,000, 17590-1-AP, Proteintech, Wuhan, China); IL-1β (1:2,000, 16806-1-AP, Proteintech, Wuhan, China); MDK (1:1,000, #DF6054, Affinity Biosciences, Jiangsu, China); P-STAT3 (1:1,000, #AF3293, Affinity Biosciences, Jiangsu, China); STAT3 (1:1,000, #AF6294, Affinity Biosciences, Jiangsu, China). GAPDH (1:5,000, 10494-1-AP, Proteintech, Wuhan, China) and β-actin (1:5000, 20536-1-AP, Proteintech, Wuhan, China) were used as the loading control. After washing the membranes three times with TBST, they were incubated with secondary antibodies (1:5,000, SA00001-2, Proteintech, Wuhan, China) for 90 min at room temperature. Protein bands were visualized using an enhanced chemiluminescence (ECL) substrate kit (Beijing Labgic Technology Co., Ltd., Beijing, China) following the manufacturer’s instructions, and the bands were quantified using ImageJ software (version 1.8; National Institutes of Health, USA).

### 2.12 Immunohistochemical staining

Paraffin-embedded tissue sections from each mouse (*n* = 5 per group) were dewaxed with xylene, dehydrated through a gradient ethanol series, and washed with phosphate-buffered saline (PBS; Gibco, USA). Following treatment with 3 % peroxide solution (Zhongshan Jinqiao Biotechnology Co., Ltd., China) for 10 min, antigen retrieval was performed using citrate buffer. Normal goat serum (Zhongshan Jinqiao Biotechnology Co., Ltd., China) was used for blocking at room temperature for 30 min. After removal of the blocking solution, the primary antibody (1:200, ab16667) was applied overnight at 4 °C. On the following day, the secondary antibody (1:1000, ab6721) was incubated at room temperature. Fresh DAB solution (Zhongshan Jinqiao Biotechnology Co., Ltd., China) was added in a humidified chamber, and slides were counterstained with hematoxylin for 5 min. After dehydration and mounting, the results were observed under a microscope. Stained slides were examined using a light microscope, and positive cells were counted in at least five randomly selected high-power fields per section.

### 2.13 Statistical analysis

All statistical analyses were performed using R software (v4.4.1) and SPSS 26.0. Experimental data were analyzed using SPSS 26.0. Measurement data were expressed as mean ± standard deviation (x̄ ± s), and normality was assessed using the Shapiro-Wilk test, while homogeneity of variance was tested with Levene’s method. A *p*-value less than 0.05 was considered statistically significant. For continuous variables, the Student’s *t*-test (for normally distributed data) or the Wilcoxon rank-sum test (for non-normally distributed data) was used. Categorical variables were compared using the chi-squared test.

## 3 Results

### 3.1 Single-cell atlas of psoriatic skin tissue

**Figure 1:**
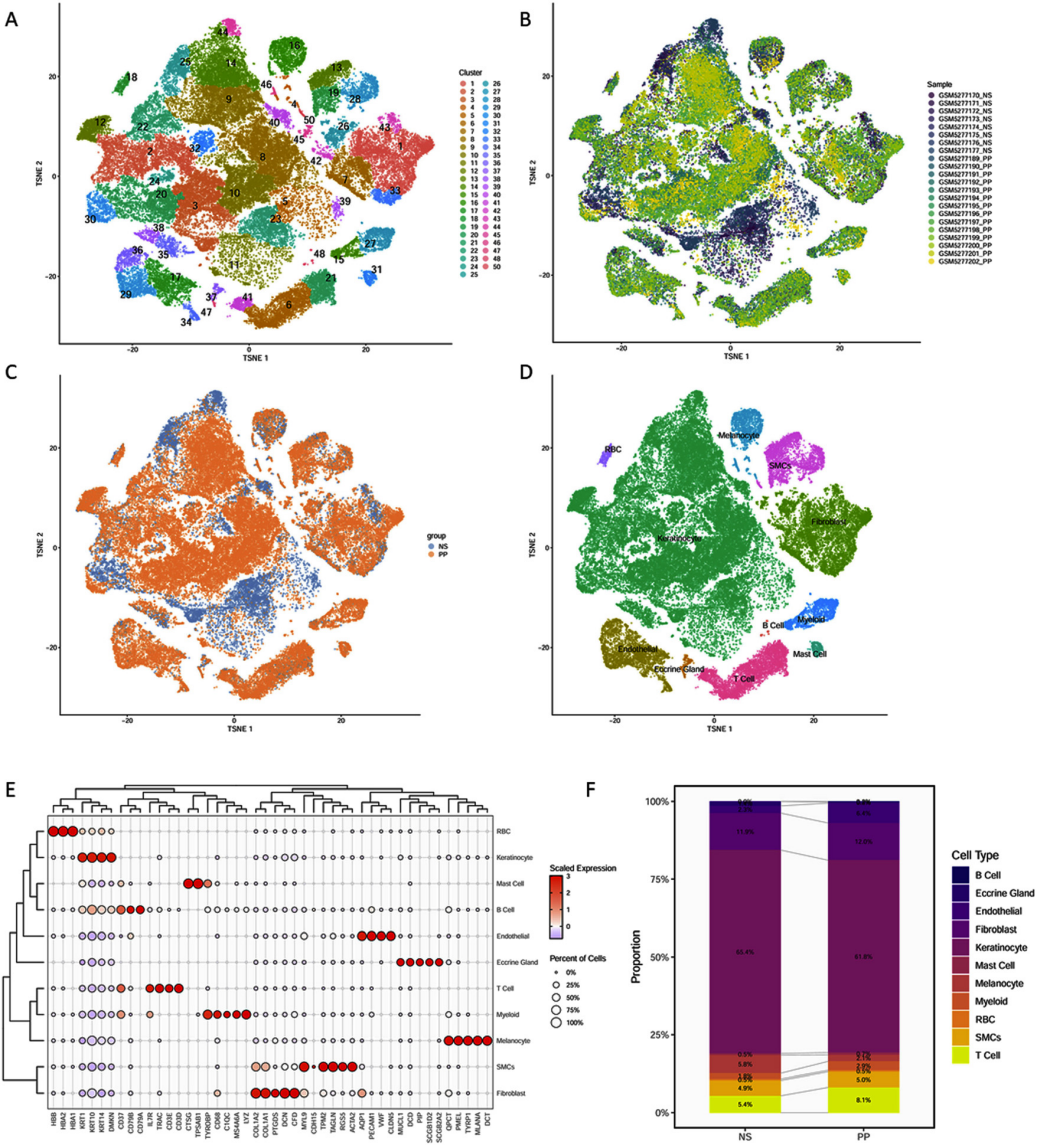
Single-cell atlas of psoriatic skin. (A) Unsupervised clustering of all cells; (B) distribution of cells by sample origin; (C) proportions of cells from different sample groups; (D) identification of all cell types; (E) bubble plot showing the expression levels of key marker genes used for cell type annotation; (F) comparison of cell composition proportions between groups. NS, normal skin; PP, psoriatic skin; RBC, red blood cells; SMCs, smooth muscle cells.

To gain a comprehensive understanding of psoriasis at the single-cell level, single-cell tissue samples were collected, followed by dimensionality reduction and clustering analyses (Figure 1A–C). Cell type annotation was performed for all clusters based on established marker genes (Figure 1D and E). The cellular composition of normal and psoriatic skin was compared to assess the impact of psoriasis on tissue cell distribution. The analysis revealed a significant increase in the proportions of endothelial cells and T cells in psoriatic tissue, rising from 2.3 % to 6.4 % and 5.4 % to 8.1 %, respectively. Conversely, melanocyte proportions decreased from 5.8 % to 2.1 %, while the distribution of other cell types remained largely unchanged (Figure 1F).

### 3.2 Functional analysis of T cells

**Figure 2:**
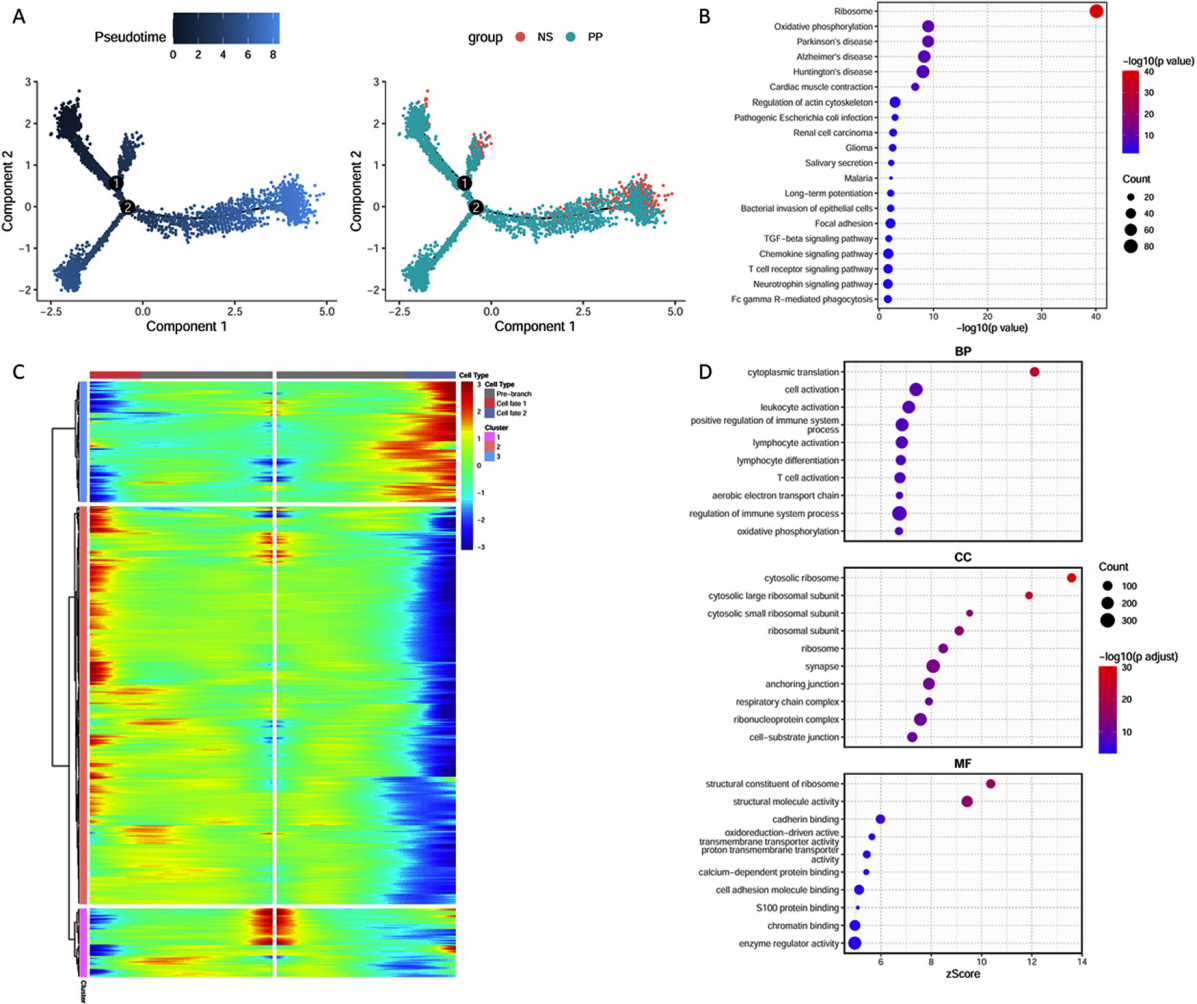
Pseudotime analysis of T cells. (A) Distribution of T cells along the pseudotime trajectory; (B) heatmap of genes with significant expression changes along the pseudotime; (C) top 20 kyoto encyclopedia of genes and genomes (KEGG) pathways enriched with genes showing significant expression changes along the pseudotime; (D) top 10 gene sets enriched in each gene ontology (GO) category. BP, biological process; CC, cellular component; MF, molecular function.

Psoriasis is recognized as an autoimmune disorder driven by abnormal T cell activation, leading to skin tissue damage and disease progression [23]. In line with this, the analysis demonstrated a significant increase in T cell infiltration in psoriatic skin compared to normal tissue. Based on this observation, further analysis was focused on T cells. Pseudotime trajectory analysis of T cells was conducted (Figure 2A), revealing a potential differentiation trajectory from normal to psoriatic tissue, with a key branching event occurring at the second node. Differential gene expression analysis at this branching point was performed, and the results were visualized in a heatmap (Figure 2B), highlighting subsets of genes specifically upregulated either before or after the bifurcation. To explore the biological functions of these branch-specific genes, KEGG pathway enrichment (Figure 2C) and GO enrichment analysis (Figure 2D) were performed. The results indicated that these genes are primarily enriched in ribosomal pathways, cytoplasmic translation, cytosolic ribosomes, and ribosomal structural components. These findings offer insights into the functional pathways associated with T cell state transitions in the context of psoriasis.

### 3.3 Cell-cell communication analysis

**Figure 3:**
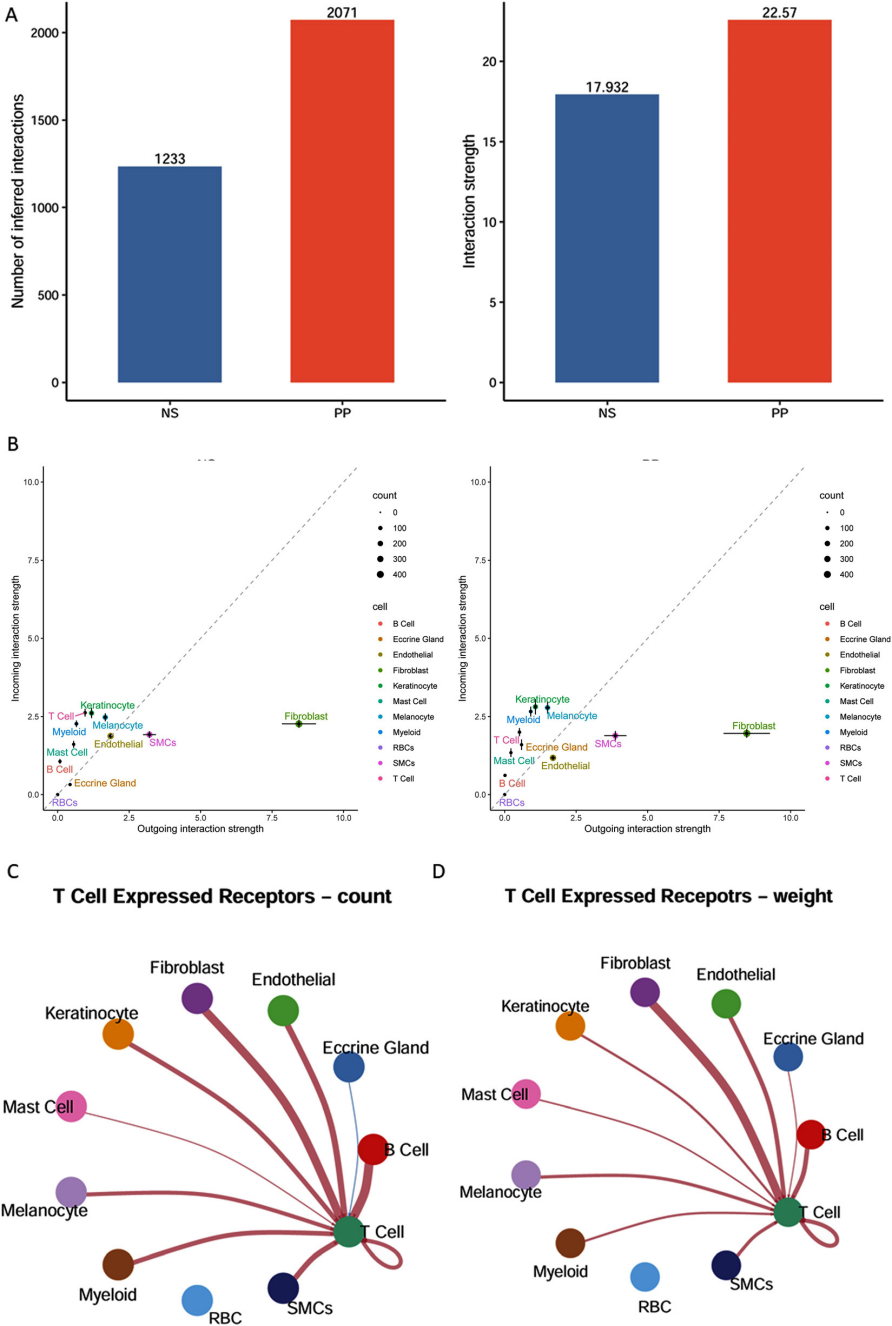
Cell-cell communication analysis. (A) Bar plots showing the number and strength of cell-cell communications in normal skin tissue and psoriatic skin tissue; (B) scatter plot of cell-cell interactions in normal versus psoriatic tissues. The *x*-axis represents the normalized number of ligand interactions secreted by each cell type, and the *y*-axis represents the normalized number of receptor interactions expressed by each cell type; (C) number of interactions between receptors expressed on T cells and ligands expressed by other cell types; (D) interaction strength between receptors on T cells and ligands from other cell types.

To explore alterations in intercellular communication in psoriasis, the number of interactions between cell populations in normal and psoriatic skin tissues was quantified (Figure 3A). The results revealed a substantial increase in both the number and overall strength of cell-cell interactions in psoriatic tissue compared to normal skin. Scatter plots were used to visualize interaction strength between each cell type and all other cell types under both conditions (Figure 3B). Notably, the interaction between T cells and fibroblasts showed the most significant change in psoriatic skin relative to normal tissue (Figure 3C and D), suggesting a potentially critical role of T cell-fibroblast crosstalk in psoriasis pathogenesis.

**Figure 4:**
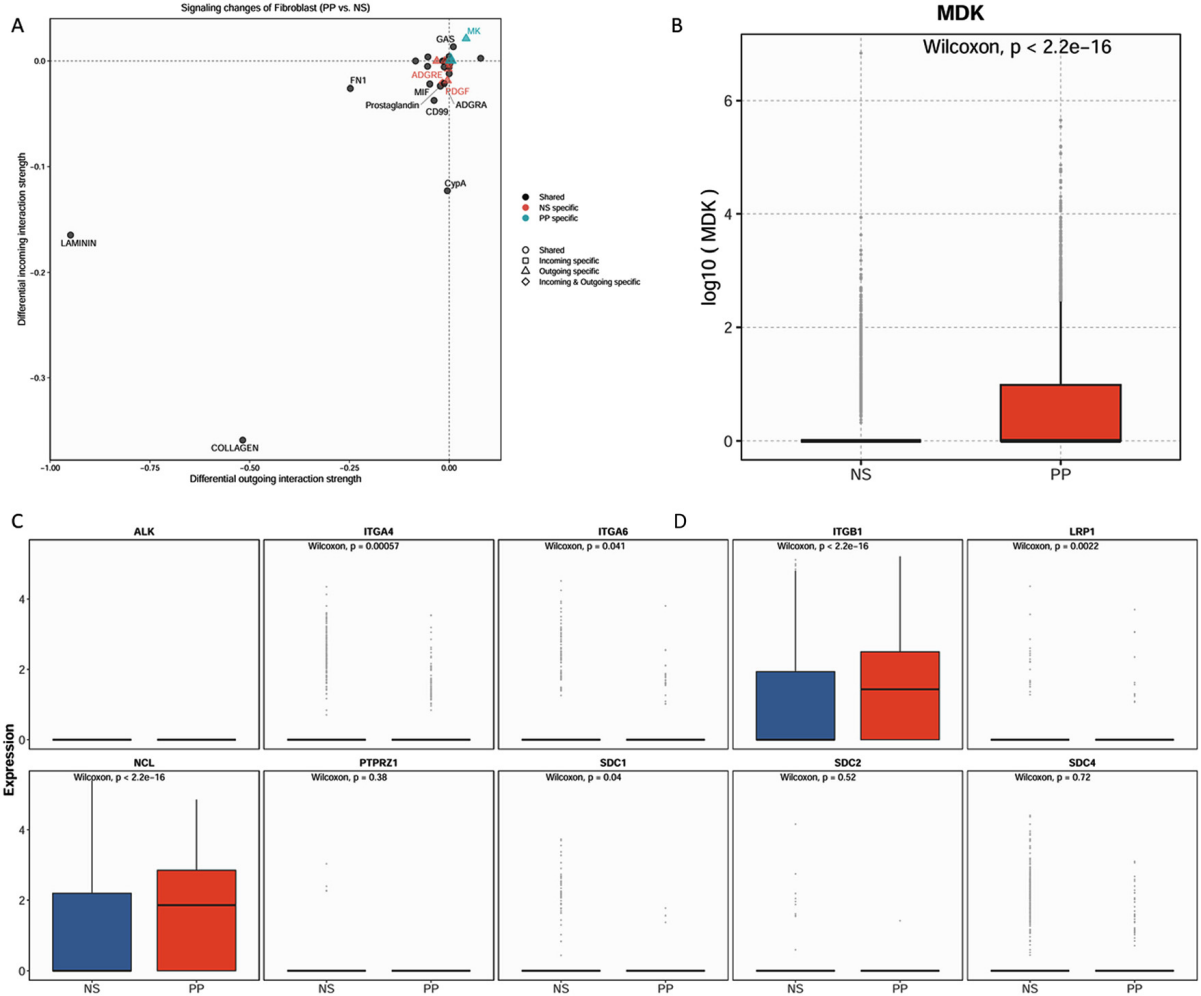
Ligand-receptor analysis. (A) Signaling pathway analysis of fibroblasts; (B) expression level of midkine (MDK) in fibroblasts; (C) expression levels of receptors in the MK signaling pathway within T cells.

Given the pronounced infiltration of T cells in psoriatic lesions (Figure 1F), fibroblasts might secrete specific chemotactic factors to recruit T cells into affected tissue. To test this hypothesis, signaling pathways enriched in fibroblasts under psoriatic conditions were analyzed. The results demonstrated a significant upregulation of the MK signaling pathway in psoriatic fibroblasts. A literature review revealed that MDK, the sole ligand in the MK signaling pathway, has established chemotactic activity [24], suggesting that fibroblasts may recruit T cells *via* MDK secretion. Subsequently, MDK expression levels were assessed in fibroblasts under both conditions (Figure 4A), which showed significantly elevated MDK expression in psoriatic fibroblasts compared to those from normal skin (Figure 4B). Additionally, the expression of MDK-associated receptors in T cells was examined (Figure 4C). Notably, expression levels of ITGB1 and NCL, two known MDK receptors, were significantly higher in T cells from psoriatic lesions. These findings support the hypothesis that fibroblasts may facilitate T cell recruitment into psoriatic skin *via* MDK-mediated chemotactic signaling.

### 3.4 Regulatory network analysis of MDK expression in fibroblasts

**Figure 5:**
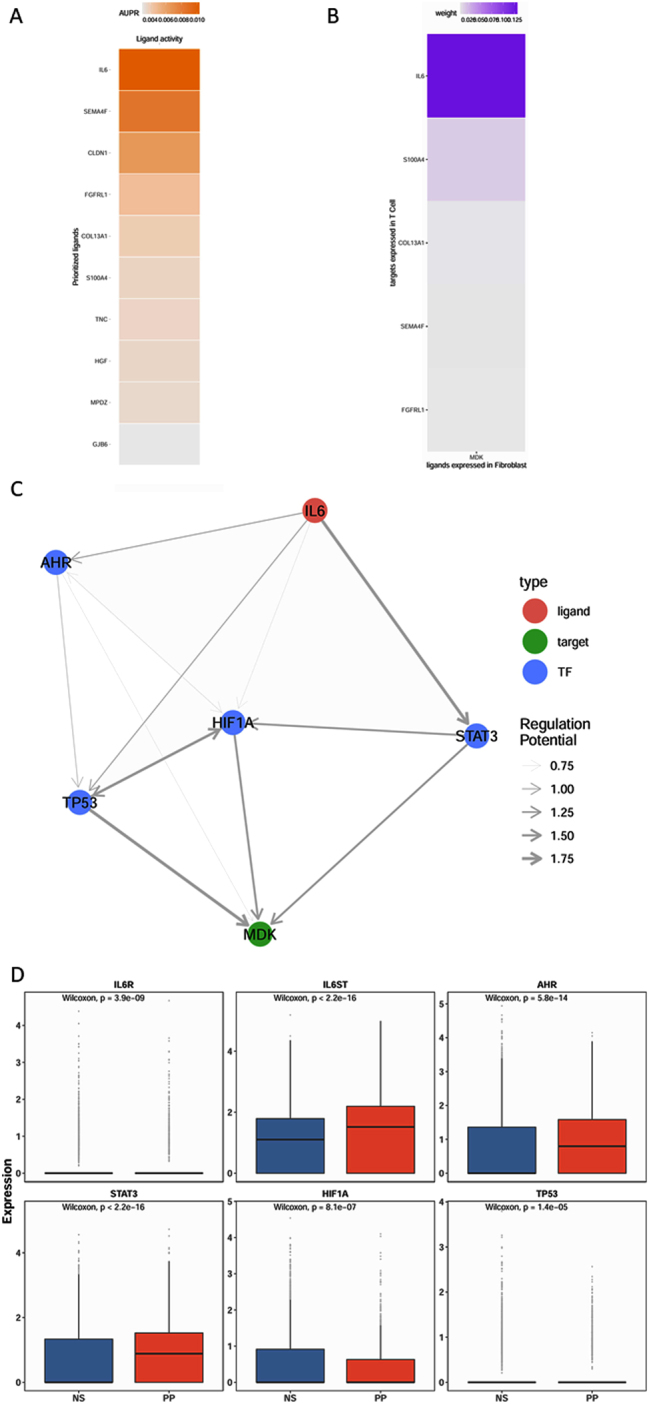
Regulatory network analysis of MDK. (A) Heatmap of ligand activity; (B) heatmap showing the likelihood of ligands regulating MDK; (C) Interleukin-6 (IL-6)-mediated regulatory network of MDK; (D) expression levels of IL6R, IL6ST, AHR, STAT3, HIF1A, and TP53 in psoriatic lesions compared with the control group. TF, transcription factor.

To investigate the upstream regulatory mechanisms governing MDK secretion by fibroblasts, a regulatory network analysis was performed using NicheNet. Candidate genes potentially regulating MDK expression were identified (Figure 5A). Further analysis of the ligand-target regulatory potential revealed that IL-6 had the highest likelihood of influencing MDK expression (Figure 5B). Next, possible transcription factors through which IL-6 could regulate MDK expression were examined (Figure 5C). The analysis indicated that IL-6 might exert its effects through several transcription factors, including STAT3, HIF1A, TP53, and AHR. Expression levels of the IL-6 receptor (IL-6R) and these transcription factors in fibroblasts were then assessed under both conditions (Figure 5D). The results showed significant upregulation of IL-6R, STAT3, and AHR in fibroblasts from psoriatic lesions, suggesting a regulatory axis where IL-6 signaling, *via* STAT3 and AHR, promotes MDK expression in psoriatic fibroblasts.

**Figure 6:**
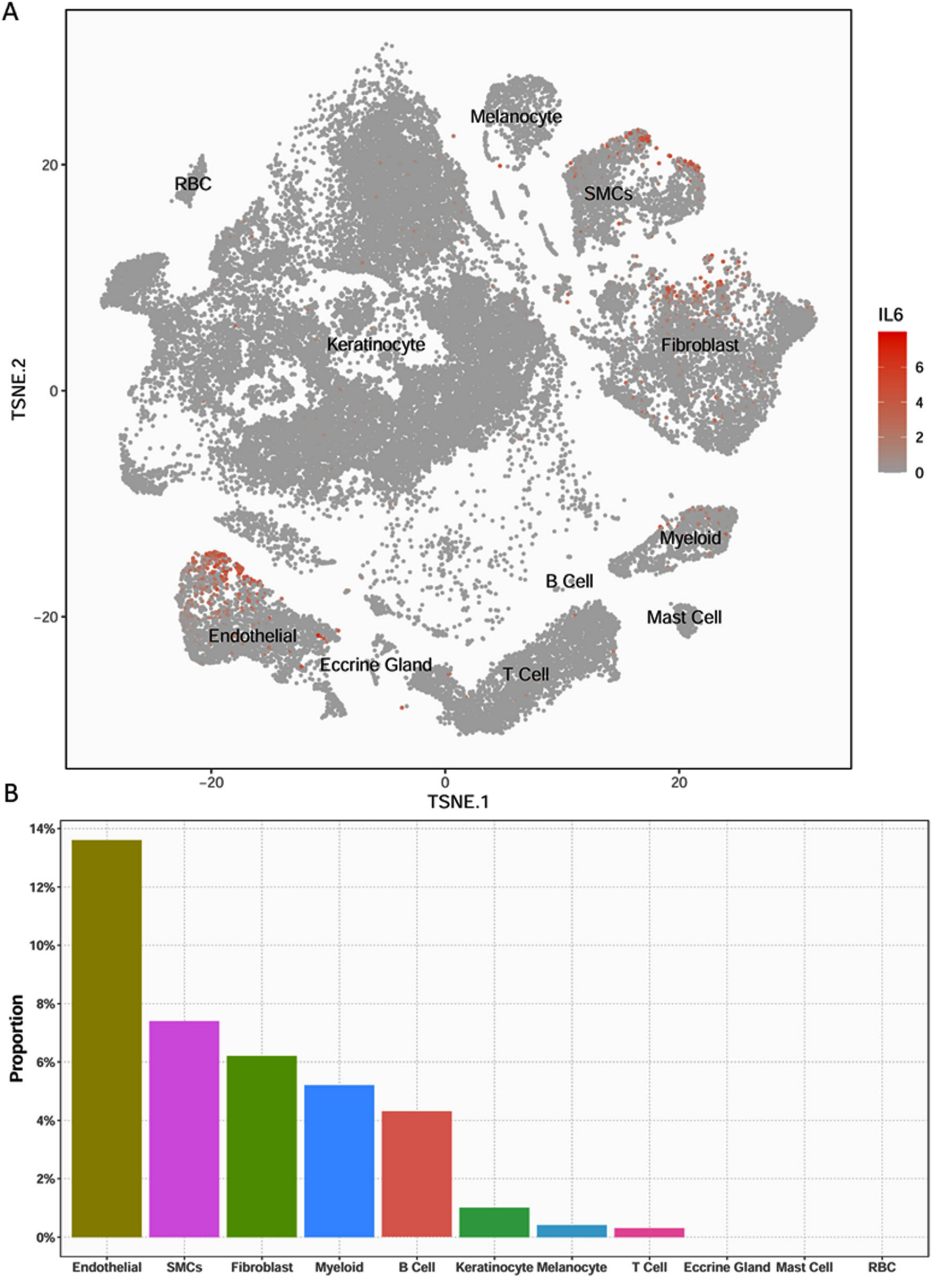
Cellular sources of IL-6. (A) T-distributed stochastic neighbor embedding (t-SNE) plot showing the expression distribution of IL-6 across different cell types; (B) proportion of IL-6-positive cells in various cell types.

Finally, the potential cellular sources of IL-6 were investigated. The t-SNE plot indicated that endothelial cells were the primary source of IL-6 expression (Figure 6A). To quantify this, the proportion of IL-6-positive cells within each cell type was analyzed (Figure 6B). Approximately 14 % of endothelial cells expressed IL-6, followed by vascular smooth muscle cells and fibroblasts, where only about 7 % of cells expressed IL-6. These results suggest that endothelial cells are the predominant source of IL-6 in psoriatic skin lesions.

### 3.5 Drug-gene interaction analysis

**Figure 7:**
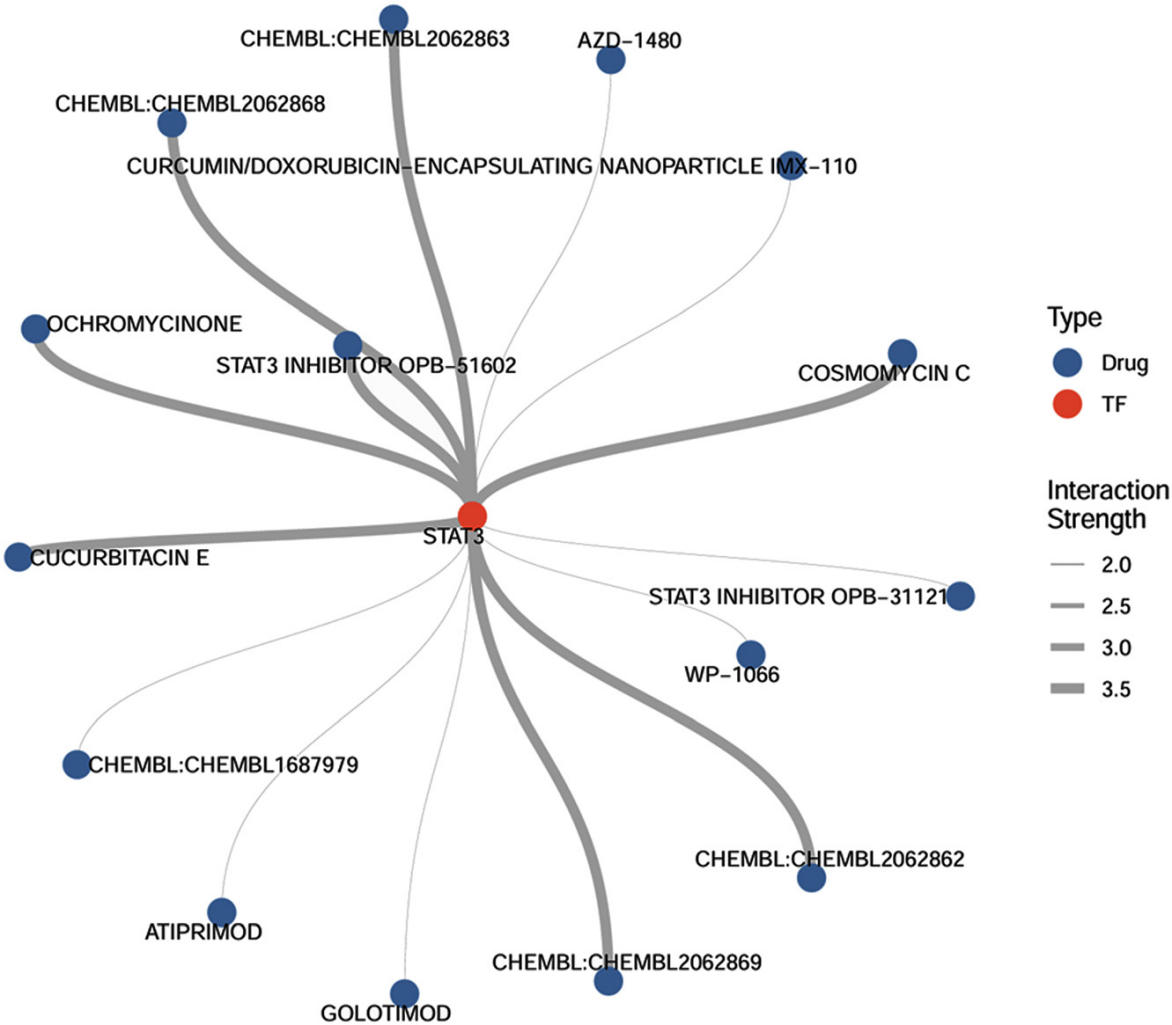
Drug-gene interaction analysis. Line thickness represents interaction strength. TF, transcription factor.

Given that IL-6 is most likely to regulate MDK expression through STAT3, the next step was to identify potential drugs capable of modulating STAT3. All drug-gene interaction data were downloaded from the DGIdb database and filtered for drugs with an interaction score greater than the mean value. The selected interactions were visualized using a network plot (Figure 7). The results identified several compounds, including COSMOMYCIN C, CHEMBL2062862, CHEMBL2062869, and CUCURBITACIN, as potential regulators of STAT3 expression. These results suggest that targeting STAT3 with these agents may modulate fibroblast-derived MDK secretion and influence T cell infiltration in psoriatic skin lesions.

### 3.6 Therapeutic effects of CuE in a murine psoriasis model

**Figure 8:**
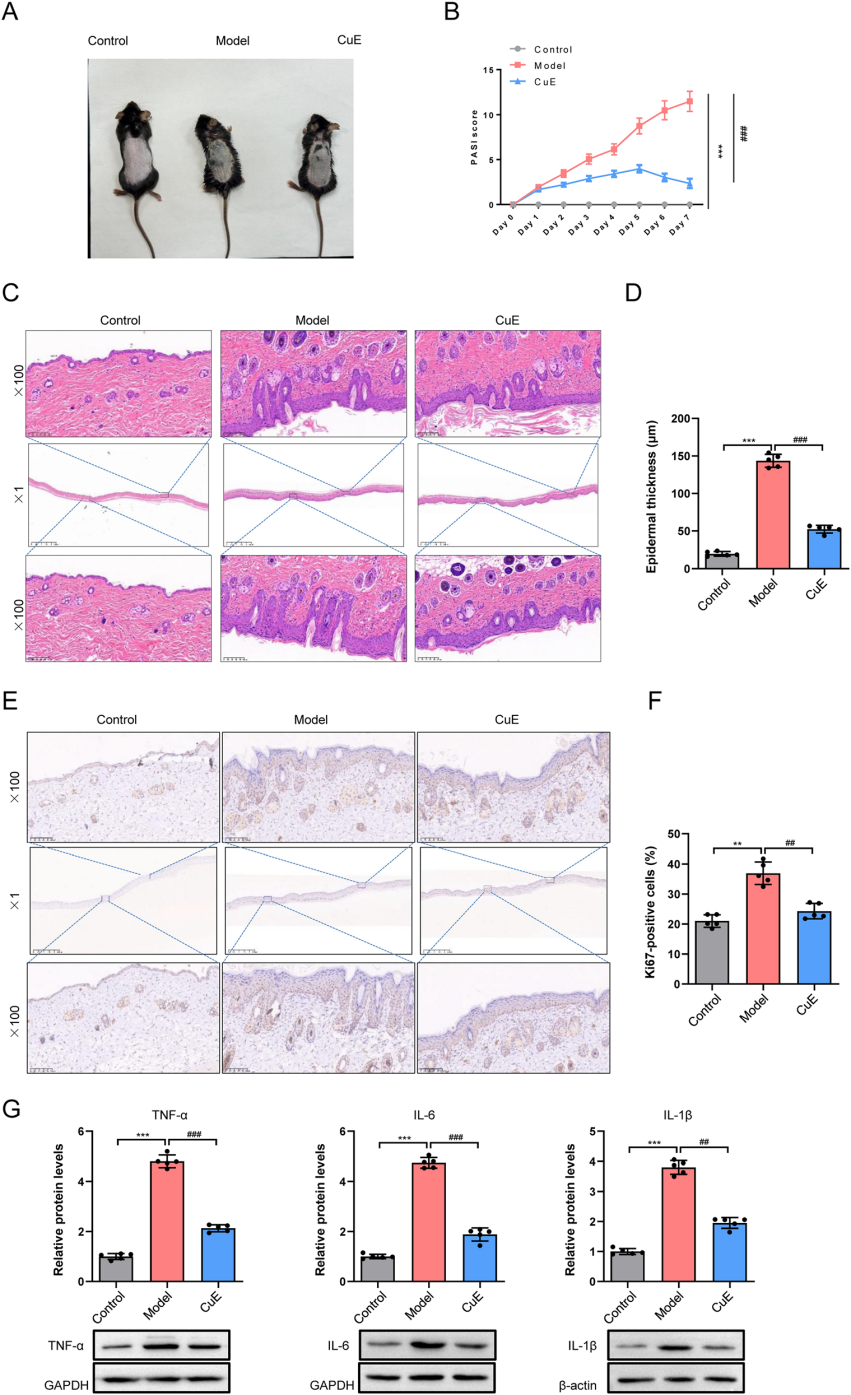
CuE alleviates IMQ-induced acute psoriasis in mice. (A) Photographs showing representative changes in mouse dorsal skin lesions; (B) psoriasis area and severity index (PASI) scores assessing psoriasis severity; (C) hematoxylin and eosin (H&E) staining showing histopathological changes in skin lesions. The overview images were captured at ×1 (scale bar 0.5 µm) and high-power images at ×200 (scale bar 20 µm); (D) quantification of epidermal thickness at lesion sites (μm); (E) immunohistochemistry (IHC) for Ki-67 expression; (F) number of Ki-67-positive cells in the epidermis. The overview images were captured at ×1 (scale bar 0.5 µm) and high-power images at ×200 (scale bar 20 µm); (G) western blot analysis of TNF-α, IL-6, and IL-1β protein levels. ^**^ *p* < 0.01, ^***^ *p* < 0.001 versus control group; ^##^ *p* < 0.01, ^###^ *p* < 0.001 versus model group.

CuE, known for its anti-inflammatory, antioxidant, and immunomodulatory properties, has shown promise as a novel therapeutic agent. Among various cucurbitacins, CuE is one of the most extensively studied for its effects on immune responses [25], [26], [27]. However, its role and therapeutic effects in psoriasis remain unexplored. Therefore, this study aimed to evaluate the efficacy of CuE in a murine model of psoriasis-like dermatitis. As shown in Figure 8A, mice in the model group developed characteristic psoriasis-like lesions, including scaly plaques and erythema on the dorsal skin. In contrast, CuE treatment markedly alleviated these clinical symptoms. PASI scores (Figure 8B) were significantly lower in the CuE-treated group compared to the model group (*p* < 0.001), indicating a notable reduction in disease severity. H&E staining (Figure 8C) revealed a thin epidermal layer with no capillary dilation or inflammatory cell infiltration in the control group. In contrast, in the model group, the stratum corneum exhibited parakeratosis, the spinous layer was thickened, and dermal structures extended into deeper layers. CuE treatment significantly reduced epidermal thickness (*p* < 0.001) (Figure 8D), suggesting attenuation of hyperproliferative pathology. Immunohistochemistry results (Figure 8E and F) showed markedly increased staining intensity and a higher number of Ki-67-positive cells in the model group compared to the control (*p* < 0.01), indicating enhanced keratinocyte proliferation. CuE treatment significantly reduced the number of Ki-67-positive cells (*p* < 0.01), suggesting an inhibitory effect on epidermal hyperplasia. Western blot analysis (Figure 8G) revealed that the expression levels of TNF-α, IL-6, and IL-1β were significantly elevated in the skin tissues of psoriatic mice (*p* < 0.001). Following CuE treatment, the expression of these pro-inflammatory cytokines was markedly reduced compared to the model group (*p* < 0.01, *p* < 0.001). These cytokines – TNF-α, IL-6, and IL-1β – are key mediators in psoriasis pathogenesis. The downregulation of their expression after CuE treatment supports its potential in mitigating inflammatory responses in psoriasis.

### 3.7 CuE inhibits STAT3/MDK pathway activity

**Figure 9:**
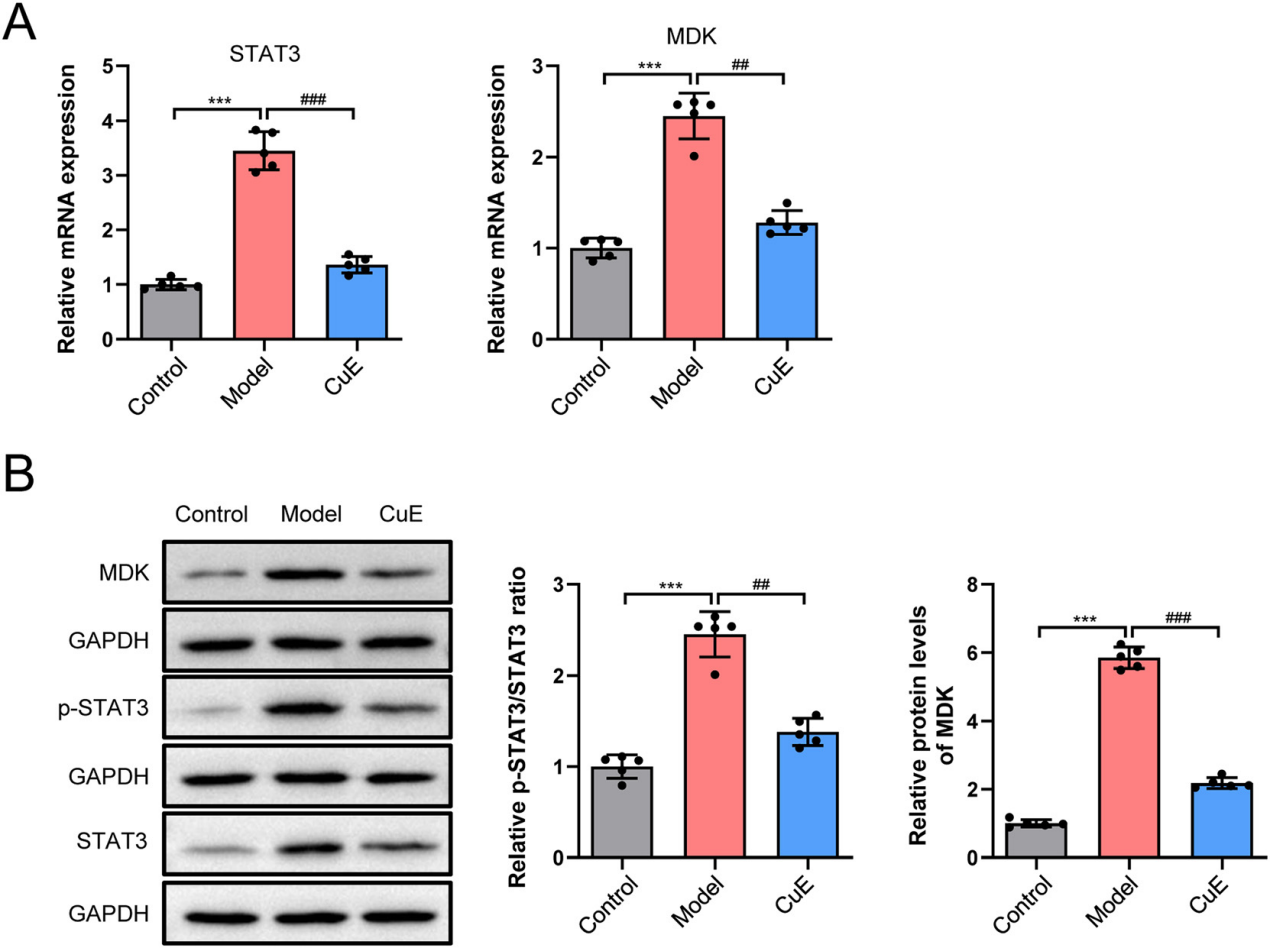
CuE inhibits STAT3/MDK pathway activity. (A) Transcription 3 (STAT3) and midkine (MDK) expression analysis by RT-qPCR; (B) Western blot analysis showing the protein expression levels of transcription 3 (STAT3), phosphorylated STAT3 (P-STAT3), and midkine (MDK). ^***^ *p* < 0.001 versus control group; ^##^ *p* < 0.01, ^###^ *p* < 0.001 versus model group.

RT-qPCR analysis (Figure 9A) demonstrated that the mRNA expression levels of STAT3 and MDK were significantly upregulated in the model group (*p* < 0.001). CuE treatment effectively reduced the expression of both STAT3 and MDK at the mRNA level (*p* < 0.01, *p* < 0.001). Consistently, Western blot analysis (Figure 9B) revealed a marked increase in the P-STAT3/STAT3 ratio and MDK protein expression in psoriatic skin tissues (*p* < 0.001). CuE treatment significantly suppressed the P-STAT3/STAT3 ratio and MDK protein level compared to the model group (*p* < 0.01, *p* < 0.001).

## 4 Discussion

This study systematically investigated the mechanisms driving increased T cell infiltration in psoriatic skin using integrated single-cell transcriptomics, pseudotime trajectory analysis, and cell-cell communication profiling. The results revealed a marked increase in T cell proportions in psoriatic lesions compared to normal skin, accompanied by significant changes in cell-cell interaction patterns. Notably, fibroblasts in psoriatic tissues exhibited elevated expression of MDK, a chemokine-like ligand involved in T cell recruitment *via* the MK signaling pathway. Additionally, regulatory network analysis identified IL-6, primarily secreted by endothelial cells, as a potential upstream regulator of MDK expression through STAT3 and AHR activation in fibroblasts. Several small-molecule agents targeting STAT3 were identified, with CuE selected for *in vivo* validation. Mouse experiments confirmed that CuE alleviates psoriasis-like symptoms by inhibiting the STAT3/MDK pathway, offering novel insights into potential therapeutic strategies for psoriasis.

Our findings align with previous studies that establish T cells as central mediators in psoriasis pathogenesis by driving keratinocyte hyperproliferation and sustaining chronic inflammation [14], 16], 17]. However, while earlier research has primarily focused on keratinocyte-T cell interactions and IL-17/IL-23 axis signaling [6], 19], few have addressed the cellular and molecular mechanisms underlying T cell recruitment into psoriatic tissues. Relevant studies have indicated that T cell infiltration in psoriatic tissues involves multiple molecular signaling pathways. IL-6–STAT3 signaling is well-established for supporting Th17 differentiation and cooperating with IL-23 signaling to maintain Th17 effector functions. Activated Th17 cells produce IL-17A and IL-22, which act on keratinocytes and stromal cells, fostering a feed-forward loop of cytokine production and chemotaxis. In our model, IL-6, secreted by endothelial cells, activates STAT3 and AHR in fibroblasts, driving MDK secretion that attracts T cells. Thus, the IL-6–STAT3–MDK axis operates in parallel with the IL-23/IL-17 pathway by contributing to T cell recruitment (chemotaxis) rather than solely T cell polarization. This provides a stromal-derived mechanism to explain how inflammatory T cells are retained and expanded within lesions, even in the presence of canonical Th17-polarizing cytokines. Using single-cell resolution and cell communication analysis, this study highlights a previously underappreciated role of fibroblasts in modulating T cell infiltration through the MK pathway. The identification of MDK as a critical chemotactic factor produced by psoriatic fibroblasts introduces a new dimension to understanding the dermal immune microenvironment. While MDK has been implicated in inflammatory diseases and tumor immunity [28], its role in psoriasis was previously undefined. Our finding that MDK receptors, ITGB1 and NCL, are upregulated in T cells from psoriatic lesions further supports the biological relevance of this pathway in T cell recruitment.

Additionally, our data highlight endothelial cells as the primary source of IL-6 in psoriatic skin, corroborating earlier reports of vascular abnormalities and endothelial activation in psoriasis [2], 29]. Further regulatory network analysis showed significant upregulation of IL-6R, STAT3, and AHR in fibroblasts from psoriatic lesions. These findings suggest that fibroblasts in the psoriatic inflammatory microenvironment exhibit heightened responsiveness to IL-6 signaling, which may lead to aberrant activation of downstream MDK expression. The upregulation of STAT3 and AHR – both well-recognized inflammation-associated transcription factors – likely amplifies IL-6 signaling effects in fibroblasts, promoting MDK secretion and enhancing T cell recruitment and infiltration [30], 31]. This observation is consistent with prior studies implicating the IL-6/STAT3 axis in regulating immune cell infiltration within chronic inflammatory and tumor microenvironments [32], 33]. The animal experiments further supported these conclusions. In the psoriatic mouse model, both the STAT3 signaling pathway and MDK were abnormally activated. It is well-established that hyperactivation of STAT3 promotes keratinocyte proliferation and the secretion of pro-inflammatory cytokines, such as IL-6 and TNF-α, while MDK exacerbates inflammatory infiltration and abnormal tissue proliferation. These pathological changes are directly linked to the hallmark features of psoriasis, including epidermal thickening and inflammatory responses, indicating that STAT3 and MDK act as key drivers of disease progression.

Following CuE treatment, both STAT3 pathway activity (as evidenced by the reduced P-STAT3/STAT3 ratio) and MDK expression were significantly suppressed. Our previous findings also confirmed that CuE downregulates the expression of inflammatory cytokines such as TNF-α and IL-6. These molecular changes correlate with observed improvements in psoriasis-like symptoms, including reduced inflammation and inhibited epidermal hyperplasia. These results suggest that CuE exerts therapeutic effects in psoriasis by modulating the STAT3/MDK signaling pathway, thereby alleviating disease-related symptoms. However, cucurbitacins are known to influence multiple cellular processes, including cytoskeletal dynamics and JAK/STAT signaling, and may exhibit toxicity at higher doses. While our data demonstrate the suppression of P-STAT3 and MDK in the IMQ model, potential off-target effects remain a possibility. Future studies should include dose-response and selectivity assays, cell-type–specific targeting approaches, and ADME/toxicity evaluations to better define CuE’s therapeutic window and its translational potential.

This study has several strengths. First, the use of a comprehensive single-cell transcriptomic dataset enabled high-resolution dissection of cellular heterogeneity and intercellular interactions. Second, the integration of pseudotime analysis, cell communication profiling, and regulatory network inference provides a multidimensional perspective on immune-stromal crosstalk in psoriasis. Notably, our finding that ribosomal and translation pathways are enriched at the trajectory branching point likely reflects a metabolic and activation switch, with activated T cells upregulating ribosome biogenesis and global protein synthesis to support cytokine production and proliferation as they transition toward the psoriatic lesion phenotype. Third, the identification of candidate drugs targeting STAT3 presents translational potential for modulating fibroblast-mediated T cell recruitment.

Despite the promising results, several limitations should be acknowledged. Specifically, the therapeutic effects of CuE were evaluated only in a single imiquimod-induced mouse model, which, although widely used, does not fully recapitulate the complexity of human psoriasis.

This study reveals a novel fibroblast-mediated mechanism driving T cell infiltration in psoriasis through the IL-6-STAT3-MDK axis. CuE treatment effectively attenuates this pathway and improves disease features, highlighting its therapeutic potential and positioning intercellular signaling as a promising target for future psoriasis interventions.

## Supplementary Material

Supplementary Material

Supplementary Material
