# Supplementary figures and images for: Dissecting the molecular mechanisms of T cell infiltration in psoriatic lesions via cell-cell communication and regulatory network analysis
Source: Open Life Sci. 2025 Dec 31;20(1):20251231. doi: 10.1515/biol-2025-1231 (PMC13011611; doi:10.1515/biol-2025-1231)

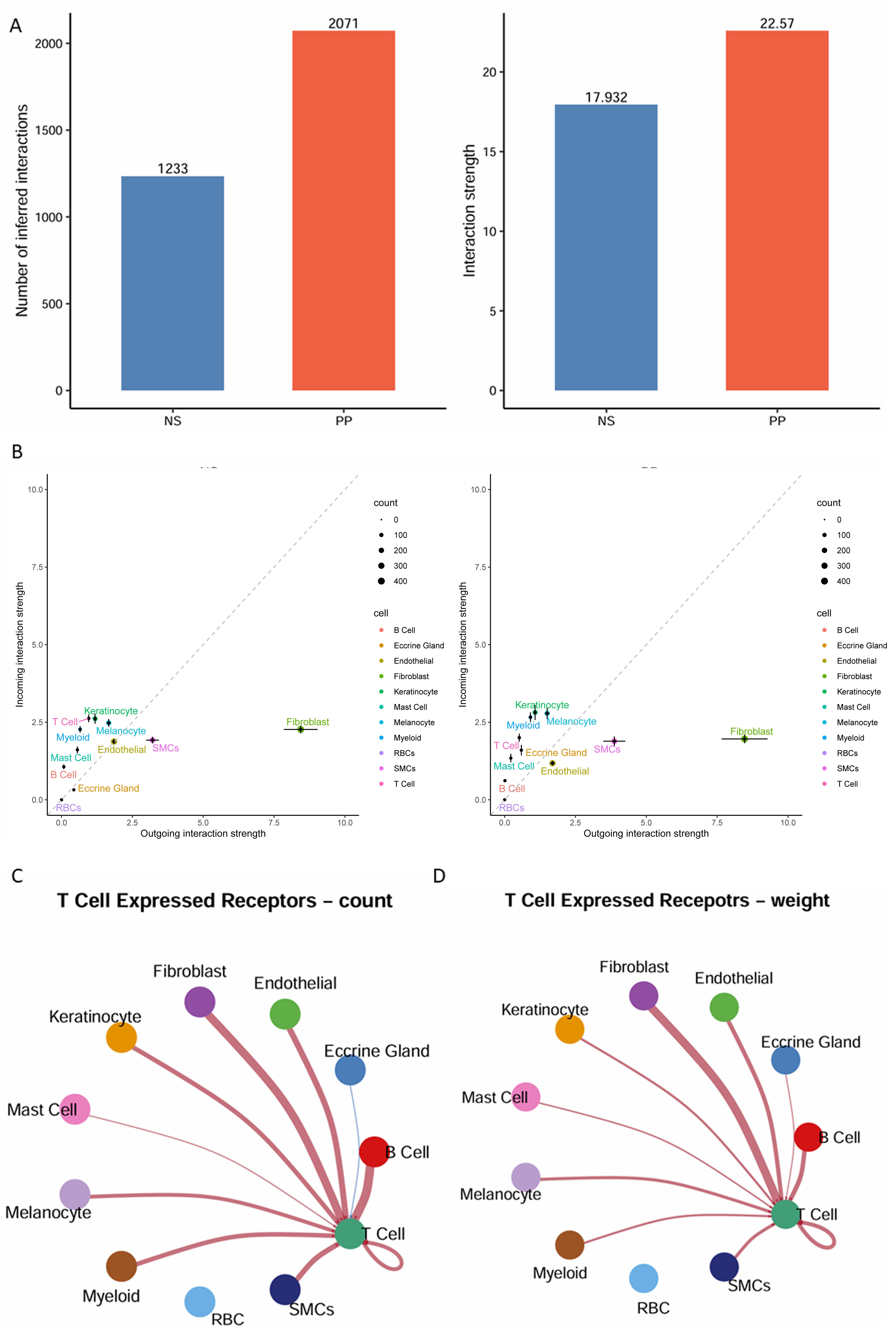

Supplement: Supplementary file 1 — Supplementary Material [file j_biol-2025-1231_suppl_001.zip › j_biol-2025-1231_suppl_001.tif]

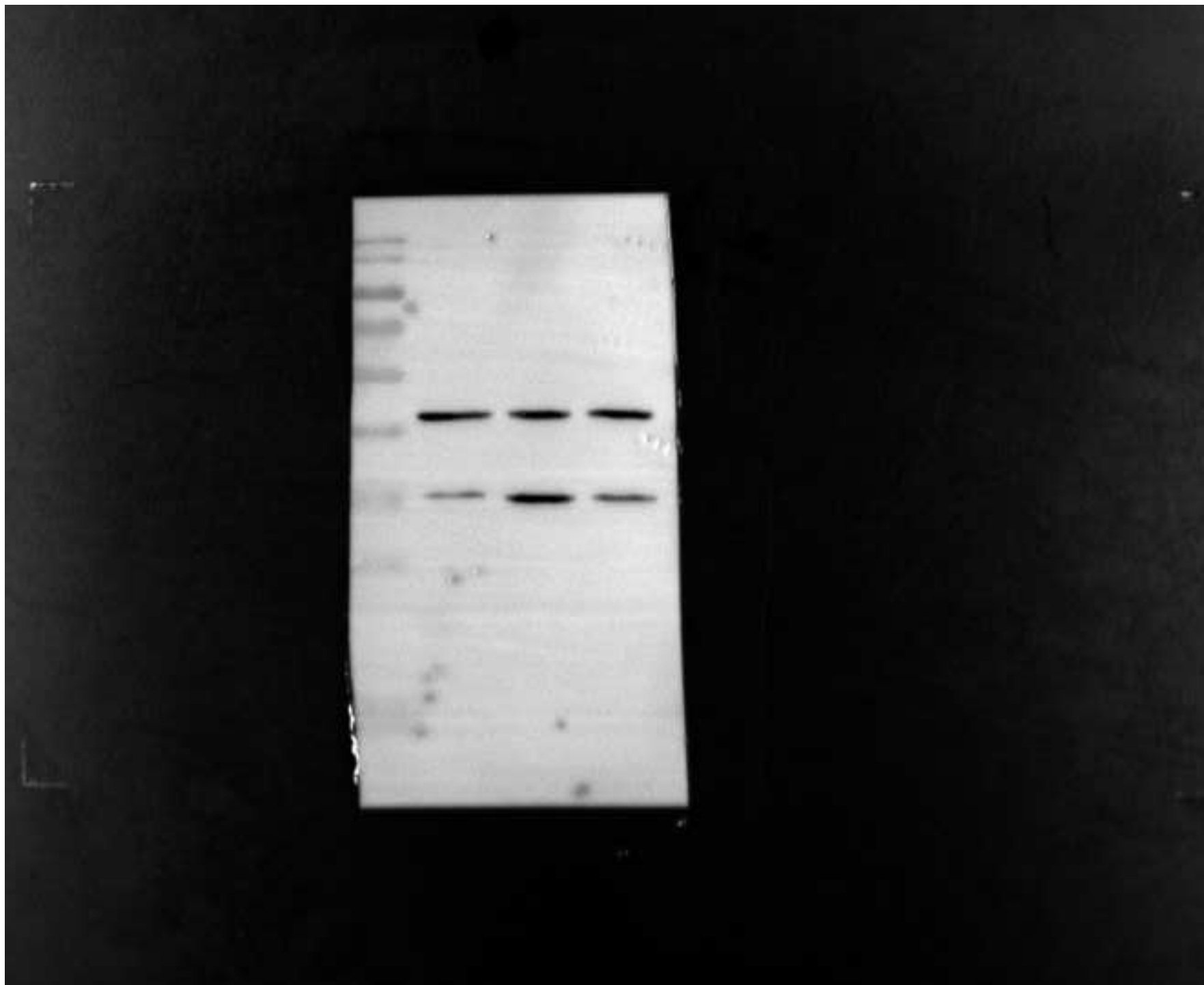

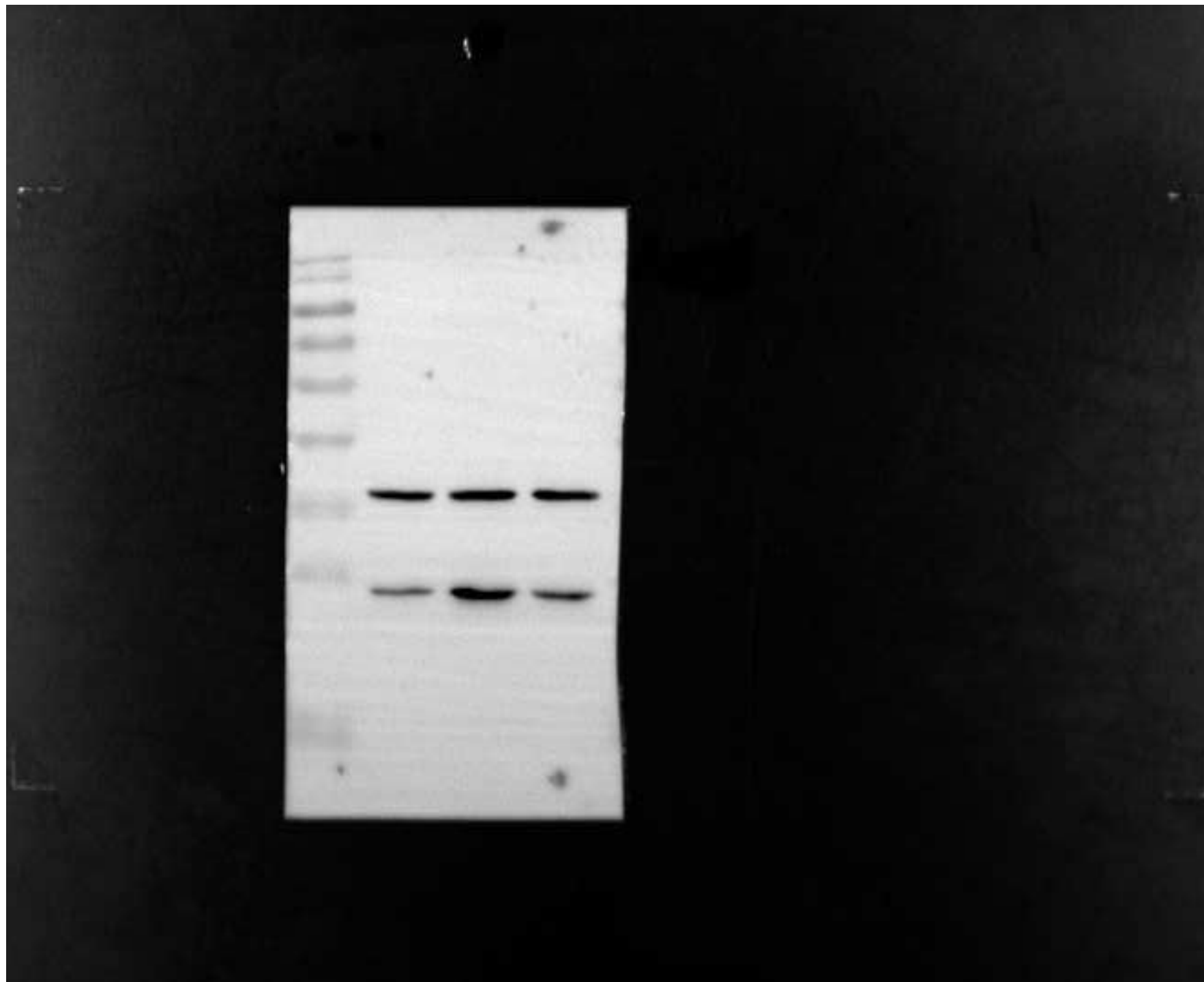

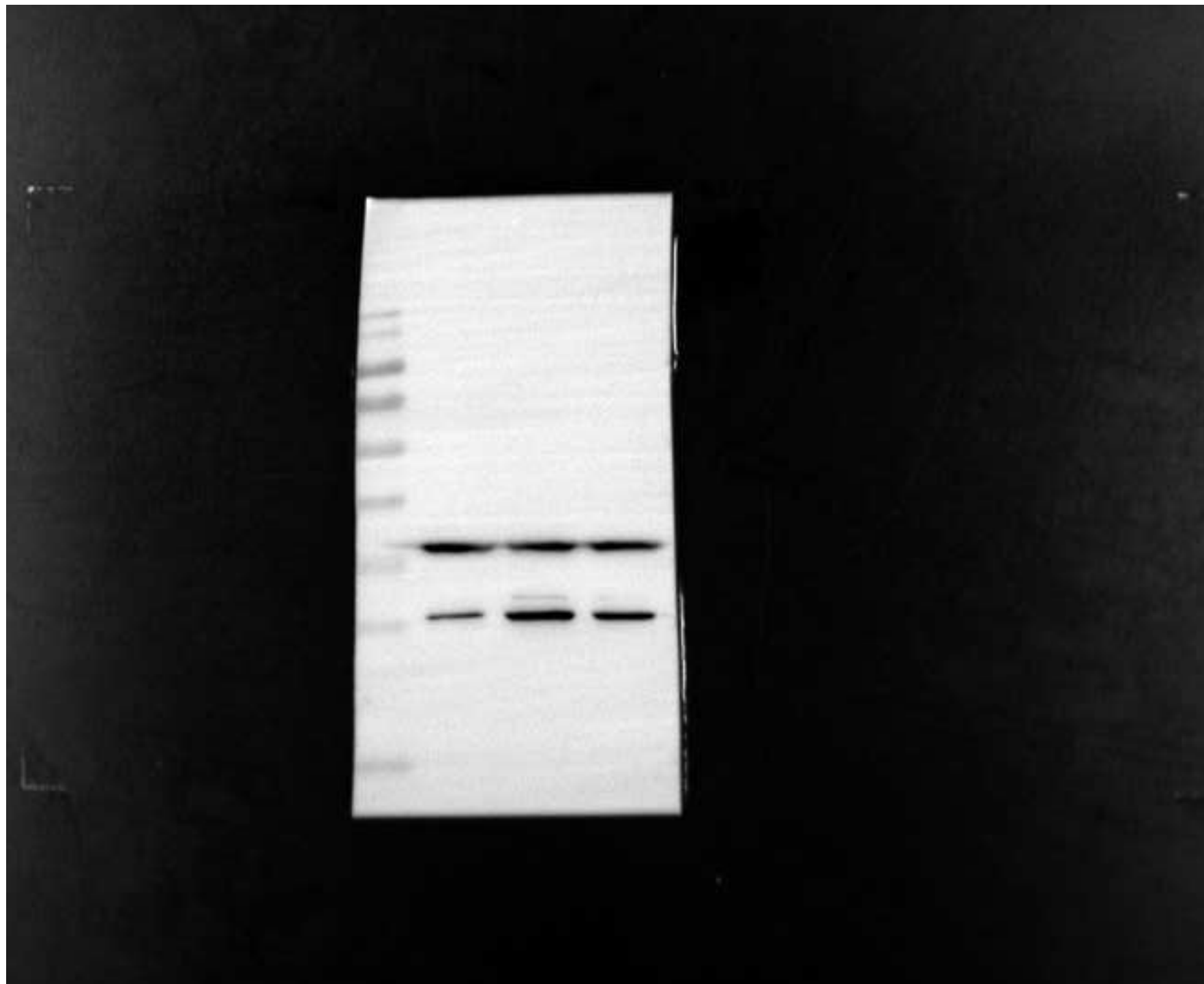

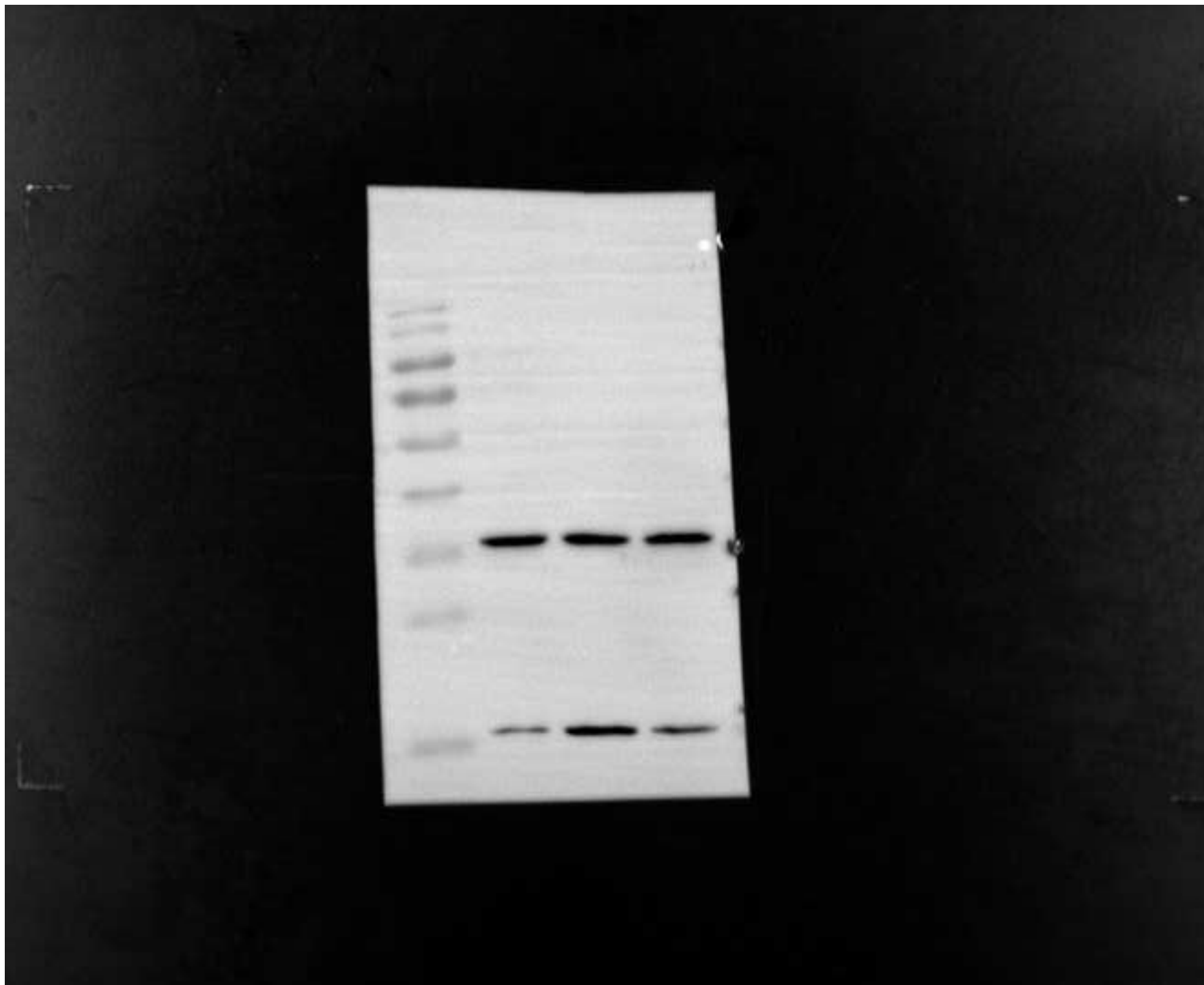

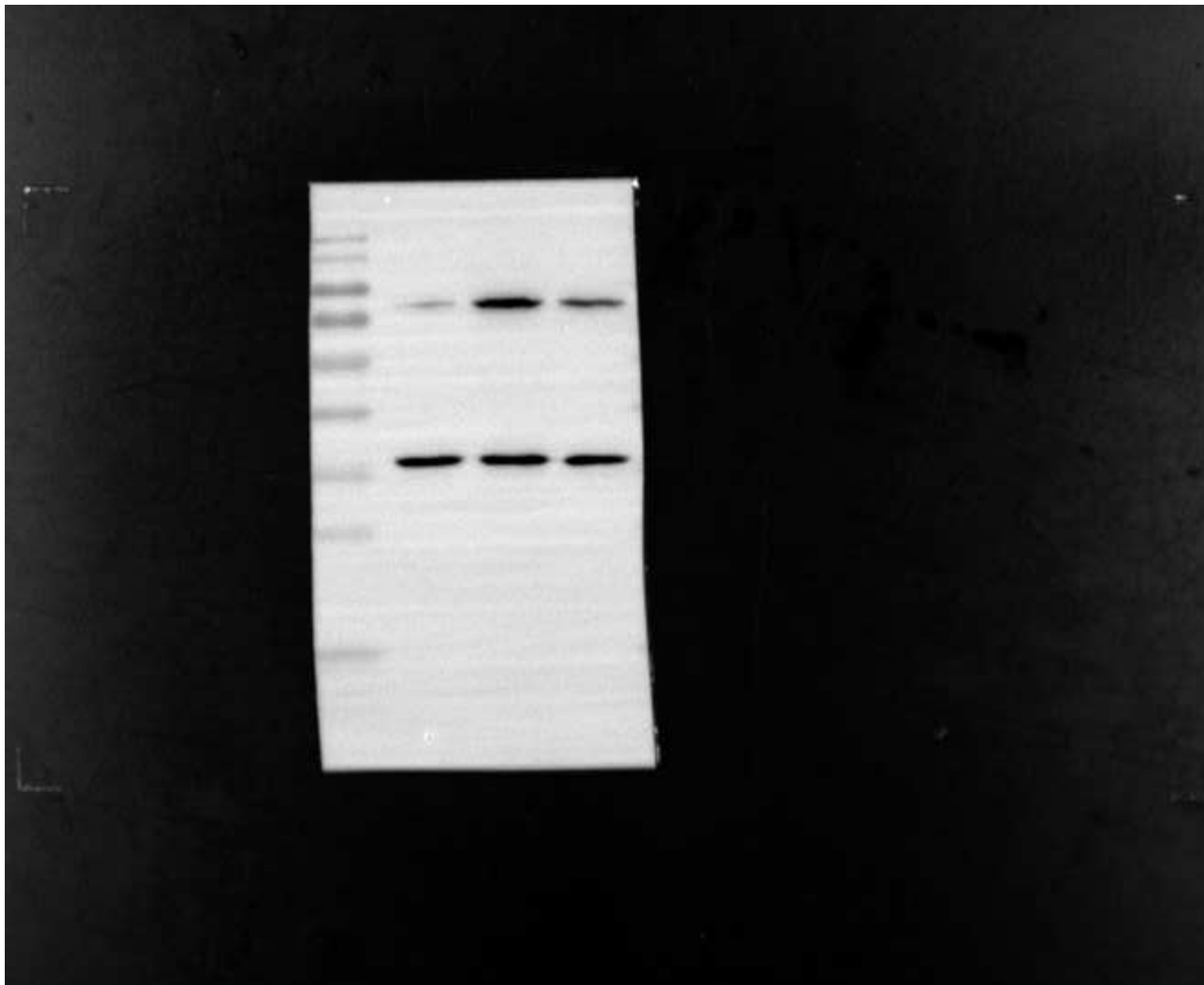

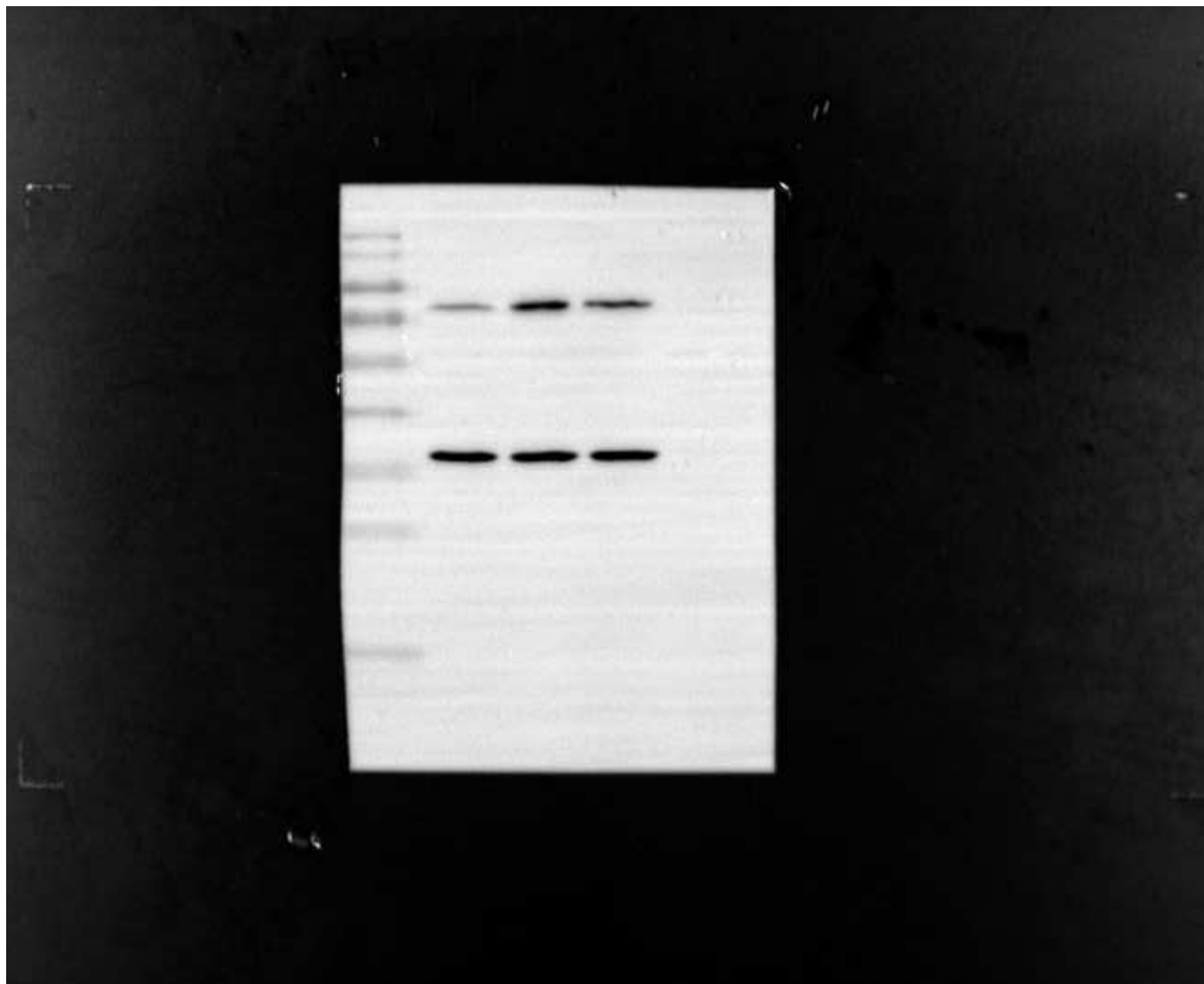

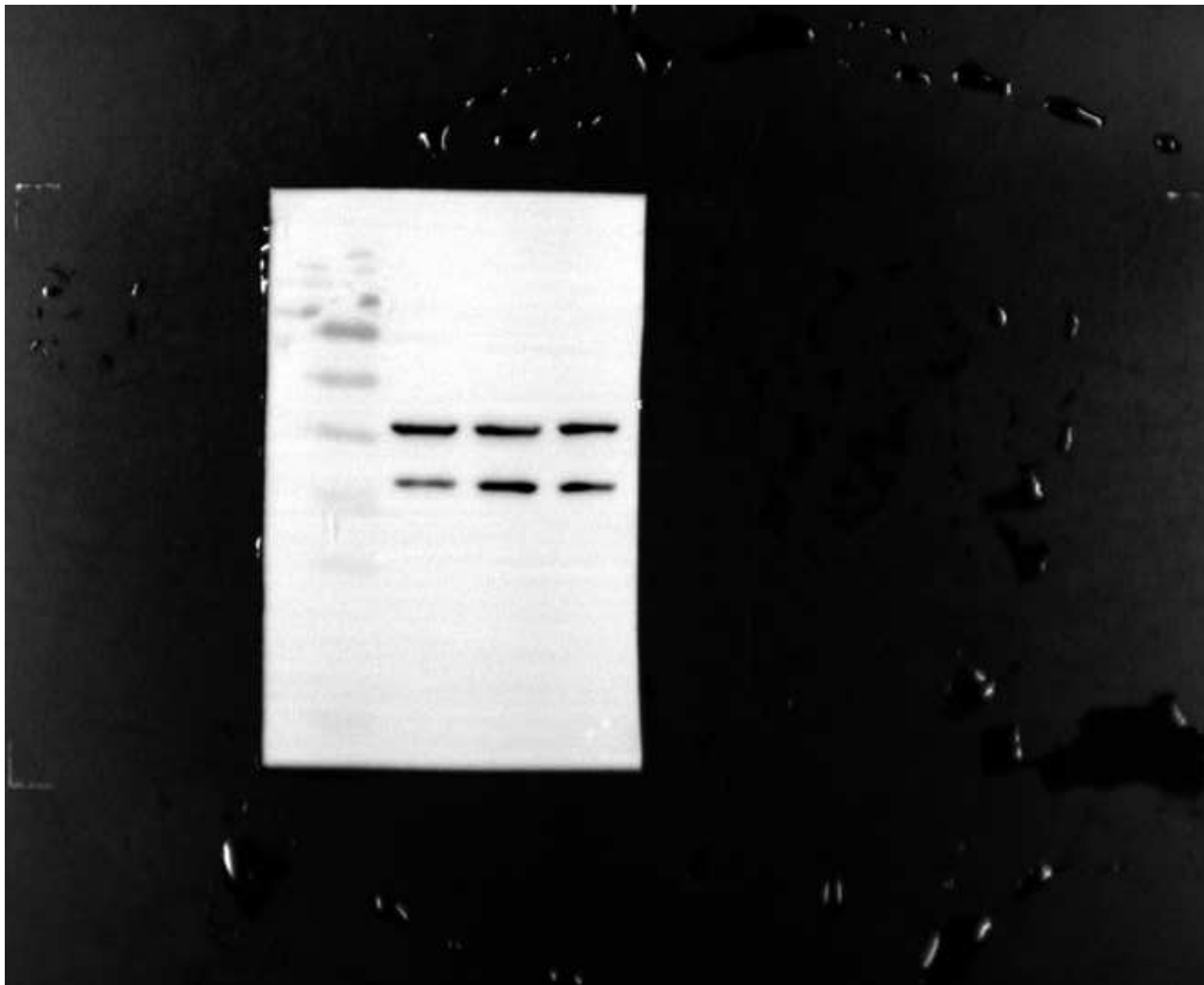

Supplement: Supplementary file 2 — Supplementary Material [file j_biol-2025-1231_suppl_002.pdf]
